# An Empirical Attempt to Operationalize Chasing Losses in Gambling Utilizing Account-Based Player Tracking Data

**DOI:** 10.1007/s10899-022-10144-4

**Published:** 2022-07-14

**Authors:** Michael Auer, Mark D. Griffiths

**Affiliations:** 1 neccton Ltd, Muhlgasse 23, 9900 Lienz, Austria; 2 International Gaming Research Unit, Psychology Department, Nottingham Trent University, 50 Shakespeare Street, NG1 4FQ Nottingham, UK

**Keywords:** Online gambling, Chasing losses, Session depositing, Problem gambling, Player tracking

## Abstract

In recent years, account-based player tracking data have been utilized as a potential tool to identify problem gambling online and associated markers of harm. One established marker of harm among problem gamblers is chasing losses, and chasing losses is a key criterion for gambling disorder in the most recent edition of the *Diagnostic and Statistical Manual of Mental Disorders*. Given the paucity of research with respect to chasing losses among online casino players using account-based data, the present study developed five metrics that may be indicative of chasing behavior: These were (i) within-session chasing, (ii) across-session chasing, (iii) across-days chasing, (iv) regular gambling account depletion, and (v) frequent session depositing. The authors were given access by a European online casino to raw data of all players who had placed at least one bet or wagered at least once during December 2021 (N = 16,771 players from the UK, Spain, and Sweden). Results indicated that frequent session depositing reflected chasing losses better than any of the other four metric operationalizations used. While frequent session depositing appears to be more indicative of chasing losses than the other four metrics, all the metrics provide useful information which can be used to help identify problematic gambling behavior online.

## Introduction

Internet gambling is a mode of gambling which has been facilitated by technology, including increased accessibility and availability of mobile devices such as smartphones and tablets (Lopez-Gonzalez et al., 2021). It should also be noted that internet gambling is not a type of gambling activity but a medium in which individuals gamble. The medium of the internet features many aspects that make it attractive to gamblers including easy accessibility, affordability, anonymity, convenience, immersion and dissociation qualities, facilitation of disinhibition, and interactivity (Griffiths, 2003).

Empirical research has indicated that internet gamblers appear to be younger, engage in a greater number of gambling activities, and are more likely to bet on sports (e.g., Gainsbury et al., 2013) although research has also shown that most online gamblers also gamble offline (Wardle et al., 2011). Allami et al. (2021) conducted a meta-analysis evaluating 57 risk factors across 104 worldwide gambling prevalence studies with the number of participants in the studies ranging from 5327 to 273,946. The risk factors (socio-demographic, psychosocial, gambling activity, and substance use correlates) were ranked from largest to smallest with regard to their association with problem gambling and internet gambling was the risk factor with the highest odds ratio. They also concluded that the most frequently assessed problem gambling risk factors with the highest effect sizes were associated with continuous-play forms of gambling.

In recent years, player tracking has been utilized as a potential tool to identify problem gambling by both gambling operators and researchers (Auer & Griffiths, 2013; Deng et al., 2019). For example, Ukohv et al. (2021) used predictive modeling to examine the differences between online sports bettors and online casino players. They found that gambling via desktop computers contributed positively to problem gambling-related exclusion among online casino players whereas for online sports bettors, problem gambling-related exclusion related more to the use of mobile devices (i.e., smartphones and tablets). Furthermore, the number of cash wagers per active day contributed the most to problem-gambling in the case of sports betting. For casino players, the most informative features related to problem gambling were shared between the volume of approved deposits and the duration of authentication sessions. Ukhov et al. (2021) concluded that the explanatory variables being considered contribute differently to problematic online casino players and problematic online sports bettors.

Luquiens et al. (2016) surveyed online poker players who were administered the Problem Gambling Severity Index (PGSI) and compared their responses with their actual gambling behavior data. They found that the most important risk factors for problem gambling were being male, depositing at least three times in a 12-hour period, being younger than 28 years, making any new monetary deposit during the 30-day study period (compared to those players who carried their balance forward from previous months), having a mean loss per gambling session of more than €1.7 during the 30-day study period, losing a total of more than €45 during the 30-day study period, having a total stake of more than €298 during the 30-day study period, having more than 60 gambling sessions during the 30-day study period, and multi-tabling (players gambling on multiple tables simultaneously).

Other studies have investigated the profile of players who have voluntarily self-excluded. For example, Percy et al., 2016 evaluated various machine learning techniques and found that the Random Forest method was the most accurate method in identifying self-excluders from gamblers who did not self-exclude. Braverman et al. (2012) analyzed the first month of data for a group of players who closed their account due to gambling-related problems. The characteristics of that group were frequent and intensive betting combined with high variability across wager amount and increased monetary wagering during the first month of betting. Given the increased incidence of problem gambling online, gambling operators have been using commercially available player tracking tools such as *PlayScan* (Griffiths, 2009) and *mentor* (Auer & Griffiths, 2020) to identify problem gambling (PG) using their players’ tracking data and informing their clientele about their gambling behavior.

Individuals who engage in maladaptive patterns of gambling behavior are said to have gambling disorder (Grant et al., 2017). Gambling disorder was classed as a form of behavioral addiction in the most recent (fifth) edition of the *Diagnostic and Statistical Manual of Mental Disorders* (DSM-5; American Psychiatric Association 2013). There are nine criteria and those individuals who endorse at least four of the criteria are diagnosed as having gambling disorder. There are three severity levels comprising mild (endorsing 4–5 criteria), moderate (endorsing 6–7 criteria), and severe (endorsing 8–9 criteria) (American Psychiatric Association, 2013; Grant et al., 2017). One of the nine DSM-5 criteria is ‘chasing losses’ which is defined as “after losing money gambling, often returns another day to get even” (p.585). Therefore, chasing losses refers to individuals increasing the amount of money that they gamble after they have lost their money gambling in an attempt to recoup the money lost. This behavior is almost omnipresent among individuals with gambling problems and has been identified by some as the most significant step or risk factor in the development of gambling disorder (Breen et al. 1999, Lesieur 1979).

However, the definition of chasing losses provided in the DSM-5 is arguably generic and not necessarily accurate, especially as chasing losses can occur within-session rather than ‘returning another day’. In short, the DSM-5’s criterion for chasing losses does not provide the extent and over what period time the monetary losses and subsequent gambling should be observed. Gainsbury et al. (2014) collected self-reported information about chasing losses among a sample of 10,838 Australian internet gamblers. The results showed that online casino players had a greater tendency to report chasing losses than online poker players and gamblers who reported chasing losses were more likely to hold irrational beliefs about gambling and spend more time and money gambling than those who reported that they were unaffected by previous losses.

Previous papers have claimed that chasing losses can easily be observed by gambling operators or researchers using account-based behavioral tracking data (e.g., Delfabbro et al., 2012; Griffiths & Whitty, 2010). However, only few studies (i.e., Catania & Griffiths 2021; Challet-Bouju et al., 2020; Perrot et al., 2018) have actually attempted to operationalize chasing losses using player tracking data. Catania & Griffiths (2021) compared the highest monetary deposit made by gamblers during the three-month study period to the initial monetary deposit after the player had first registered on the website. This ratio was argued by the authors to be an operational metric for chasing losses. However, the metric developed by Catania and Griffiths (2021) did not reflect short-term chasing which could potentially happen in a gambling session. Additionally, the metric also did not accurately reflect the DSM-5 criterion which simply defines chasing as increased gambling on a day after losing more on a previous day.

Perrot et al. (2018) analyzed player account data of 10,000 French online lottery players. They operationalized chasing losses as either three or more deposits within a 12-hour period or a deposit less than one hour after a previous bet. Chasing losses was one variable used in a cluster analysis which identified seven distinct clusters of online lottery players. They found a small cluster (3% of the total sample) which was characterized by a high gambling activity and which comprised a higher proportion of female players with a high probability of chasing behavior, and a large proportion of their bets comprising instant lottery games (which is unsurprising given that instant lottery games are continuous games with high event frequencies unlike most other lottery products such as bi-weekly lotto games).

In another study of French online lottery players, Challet-Bouju et al. (2020) used the same operationalization of chasing losses as Perrot et al. (2018). Challet-Bouju et al. performed a cluster analysis and identified one cluster which was characterized by medium to very high gambling activity, played a higher number of game types, and had a high number of episodes of chasing losses. However, the studies by Perrot et al. (2018) and Challet-Bouju et al. (2020) both comprised online lottery players only. Moreover, their participants could only purchase lottery tickets and/or online instant win games. Therefore, their operationalization of chasing losses and their findings are not directly applicable to other types of gamblers such as sports bettors, roulette gamblers, and slots players. Online casino products such as slots games typically have a much higher event frequency and are more elaborate with respect to audio-visual aspects. The present authors believe that the time period to observe chasing losses should be less than 12 h in the case of online casino gambling. Several other studies have used shorter time periods to study online casino gambling behavior (e.g., Auer et al., 2014; Hopfgartner et al., 2021).

Given the paucity of research with respect to chasing losses among online casino players using account-based data, the present study developed a number of new metrics using tracking data to reflect short-term chasing behavior as well as chasing on a day after losing on the previous day. To the best of the authors’ knowledge, the present study is the first to compare different metrics for chasing losses using real-world players and player tracking data. Given that the present study was necessarily exploratory, there was no specific hypothesis, other than it was expected that chasing losses would be observed to be more likely among higher involved gamblers.

## Method

The authors were given access by a European online casino to raw data of all players who had placed at least one bet or wagered at least once during December 2021. The data comprised each wager and each win as well as each deposit and each withdrawal. The data also contained the amount of money in the gambling account (balance) before and after each transaction. The authors were also given access to each player’s age and gender. The authors computed gambling sessions based on the raw data. Sessions were computed based on the timestamp of the single wagers. If two wagers were placed within 15 min of each other, the time between those two events counted as session time. If there was more than 15 min between two wagers, the time between the two events was not counted as session time.

### Metrics for chasing losses

The authors developed five different metrics for chasing losses based on the single transactions and derived sessions. These were (i) within-session chasing, (ii) across-session chasing, (iii) across-days chasing, (iv) regular gambling account depletion, and (v) frequent session depositing. These are described below.

*Within-session chasing*: The authors sorted the single wagers within each session by the timestamp. The wagers within a session were assigned a consecutive number from 1 to n_s_ where n_s_ was the number of wagers in session s. The Spearman correlation coefficient was then computed between the wagers and the consecutive numbers for each session via the formula corr_i,s_^1^(w_i,s_,t_i,s_). A positive correlation indicates that the amount of money wagered is increasing throughout a session and a negative correlation indicates that the amount of money wagered is decreasing throughout a session. The correlation can only be computed for sessions with at least three wagers. The correlation is undefined for sessions with a constant amount wagered, because the standard deviation is zero. The standard deviation of a vector where each number is the same is zero. The authors computed one average value for a player across all the correlations computed within each session using the following formula: corr_i_^2^ =avg(corr_i,s_ [w_i,s_,t_i,s_]).

*Across-session chasing*: The authors also sorted the sessions for each player by time. Each session’s monetary loss as well as amount of money wagered was then computed. Loss was computed as amount won minus amount wagered. A negative value indicates that the amount of money wagered was larger than the amount won. A positive value indicates that the amount of money won was larger than the amount wagered. The Spearman correlation between a session’s monetary loss and the next session’s amount of money wagered was then computed across all sessions for each player using the following formula: corr_i_^3^(l_i,t−1_,w_i,t_). A negative correlation indicates that a player tends to wager more money in a session if the previous session’s monetary losses were larger. A positive correlation indicates that a player tends to wager less money in a session if the previous session’s monetary losses were larger. A pair of sessions was only considered if they happened within a 24-hour period.

*Across-days chasing*: The authors sorted the number of gambling days for each player by time. Each day’s monetary loss as well as amount of money wagered was then computed. Loss was computed as amount of money won minus amount of money wagered. A negative value indicates that the amount of money wagered was larger than the amount of money won. A positive value indicates that the amount of money won was larger than the amount of money wagered. The Spearman correlation between a day’s monetary loss and the next day’s amount of money wagered was then computed across all days for each player. The same formula as ‘across session chasing’ was used, except that the monetary loss and amount of money wagered for entire days was computed. A negative correlation indicates that a player tends to wager more money on a day if the previous day’s monetary losses were larger. A positive correlation indicates that a player tends to wager less money on a day if the previous day’s monetary losses were larger. Only pairs of consecutive days were considered.

*Regular gambling account depletion*: The authors also had access to the amount of money in the gambling account before and after each transaction (also referred to as the balance). The amount of money in the gambling account after the last game of a session was computed. For each player, the authors computed the percentage of sessions when there was less than €5 in the gambling account at the end of the session. If players regularly deplete their gambling account it may be an indication of chasing and not being able to stop gambling.

*Frequent session depositing*: Finally, for each player, the authors computed the percentage of sessions with more than one monetary deposit. Depositing frequently in a session may be an indication of chasing after losses and not being able to stop gambling.

### Problem gambling risk score

The operator which provided the player tracking data uses the behavioral tracking tool *mentor*. The system’s personalized feedback was evaluated in two previous studies and both of these studies provided empirical evidence that the system is beneficial to gamblers (Auer & Griffiths, 2015, 2020). On a daily basis, the behavioral tracking tool computes gambling-related risk for every player and classifies each player who was active during the previous 30 days into one of three gambling risk categories (low-risk, medium-risk, high-risk). The risk score is based on the amount of money spent gambling, amount of time spent gambling, and more specific variables such as the number of failed deposit attempts, the number of money withdrawals which were cancelled by players, and high deposit limit-setting. The risk score takes into account up to six months’ past gambling behavior.

The authors tested whether the five metrics developed for chasing losses followed a normal distribution according to D’Agostino (1971). Nonparametric Kruskal Wallis tests were used for group comparisons (Kruskal, 1952). A multinomial regression (Böhning, 1992) was used to predict the risk score group based on the independent chasing losses variables, and *p*-values less than 1% were regarded significant. The k-means method (Likas et al., 2003) was used to perform a cluster analysis. The authors used the programming language *Python* (Van Rossum, 2007) to analyze the dataset. For the multinomial regression, the *Python* statsmodel package was used.

### Participants

A total of 16,771 players wagered money on the gambling operator’s website between December 1 and December 31 (2021) and fulfilled the aforementioned criteria for each of the five chasing losses metrics to be computed. Players were from the UK, Spain, and Sweden. The average age was 41 years (SD = 11.85) and the sample comprised 8,432 females (50.27%) and 8,339 males (49.72%).

## Results

Table 1 reports the number of players and average values for each risk category. The behavioral tracking system computed a daily risk score for each player. Players were assigned their maximum risk score from December 2021. Player age (K^2^ = 876, *p* < 0.001), number of sessions (K^2^ = 12,672, *p* < 0.001), number of playing days (K^2^ = 2,437, *p* < 0.001) and number of deposits (K^2^ = 16,597, *p* < 0.001) significantly deviated from a normal distribution.

A Kruskal-Wallis test showed there were significant different differences between the three risk scores in relation to age (K^2^ = 26.68, *p* < 0.001), number of sessions (K^2^ = 1346, *p* < 0.001), number of playing days (K^2^ = 672, *p* < 0.001), and number of deposits (K^2^ = 3581, *p* < 0.001). The percentage of females in the three risk groups was significantly different (χ^2^ = 52, *p* < 0.001). Although age was significantly different between the risk groups, there was less than one year difference between the three groups. However, it should be noted that with over 16,000 participants and numerous observations per participant, statistically significant results may or may not reflect meaningful results, and should therefore be interpreted with this in mind.

There were significantly fewer female players in the high-risk group (43%) than in the medium-risk (46%) and low-risk-group (52%). There was also a clear pattern with respect to the number of gambling sessions in the one-month study period. Players in the high-risk group had (on average) 39 sessions, players in the medium-risk group had 32 sessions, and players in the low-risk group had 20 sessions. The same descending pattern is true for the number of playing days as well as for the number of deposits. Players in the high-risk group played (on average) 15 days during the one-month study period, players in the medium-risk group played on 14 days, and players in the low-risk group played on 11 days. Players in the high-risk group deposited (on average) 25 times during the one-month study period, players in the medium-risk group deposited 14 times, and players in the low-risk group deposited four times.

**Table 1:** Mean average number of sessions, playing days, and deposits for each gambling risk score category in December 2021

Risk score	Low-risk	Medium-risk	High-risk
N	12,936 (77.13%)	2757 (16.44%)	1078 (6.43%)
Age (in years)	40.61 (11.96)	41.57 (11.72)	41.41 (10.64)
Female	52%	46%	43%
Number of sessions	20.97 (15.02)	32.00 (25.78)	39.01 (30.54)
Number of playing days	10.93 (5.4)	13.76 (6.86)	15.02 (7.02)
Number of deposits	4.11 (5.76)	14.00 (13.69)	24.57 (25.56)

The five different chasing losses metrics all significantly deviated from a normal distribution: within-session chasing (K^2^ = 116.34, *p* < 0.001), across-session chasing (K^2^ = 14.58, *p* < 0.001), across-days chasing (K^2^ = 761.14, *p* < 0.001), regular gambling account depletion (K^2^ = 1 332.37, *p* < 0.001), and frequent session depositing (K^2^ = 7908.76, *p* < 0.001).

Table 2 reports the average chasing losses values for each gambling risk category. A Kruskal-Wallis test showed there were significant differences between the three gambling risk groups in within-session chasing (K^2^ = 37.47, *p* < 0.001), regular gambling account depletion (K^2^ = 118.76, *p* < 0.001), frequent session depositing (K^2^ = 2399.78, *p* < 0.001) and across-session chasing (K^2^ = 28.49, *p* < 0.001). There were no significant differences between the three gambling risk groups in across-days chasing (K^2^ = 1.62, *p* = 0.45).

**Table 2:** Mean average numbers for the various chasing losses metrics for each risk category

Risk score	Low-risk	Medium-risk	High-risk
N	12,936 (77.13%)	2,757 (16.44%)	1,078 (6.43%)
Within-session chasing	-0.01	0.01	0.00
Across-session chasing	0.08	0.06	0.05
Across-days chasing	0.07	0.09	0.08
Gambling account depletion	69%	65%	64%
Frequent session deposits	5%	13%	18%

In order to study the effect of all five chasing losses metrics together, a multinomial regression was performed. The dependent variable was the risk score with three categories and the independent variables were the five chasing losses metrics. The pseudo R^2^ was 8.7%. The log likelihood of the model was − 10,318 and the log likelihood of the null model was − 11,296. The difference was statistically significant (*p* < 0.001).

The output in Table 3 shows each independent variable’s coefficient. The coefficients are the log odds and have to be interpreted with respect to the high-risk group which was chosen as the reference group utilizing multinomial regression. Two chasing losses metrics (i.e., regular gambling account depletion and frequent session depositing) were significant among both low-risk and medium-risk players (i.e., regular gambling account depletion and frequent session depositing). Each coefficient reports the likelihood of belonging to the low-risk or medium-risk group compared to the high-risk group. A negative coefficient means that the chance of being in the low-risk group or medium-risk group is decreasing with an increase in the independent variable. In relation to the present findings, frequent session depositing had a negative coefficient among both low-risk and medium-risk gamblers compared to high-risk gamblers (Table 3). The greater the chasing losses, the lower the chance of being a low-risk or medium risk gambler.

Increasing gambling account depletion by one unit resulted in an increase of 1.2 units in the log of the ratio between the probability of being low-risk vs. high-risk. Increasing frequent session depositing by one unit resulted in a decrease of -7.4 units in the log of the ratio between the probability of being low-risk vs. high-risk. Increasing gambling account depletion by one unit resulted in an increase of 0.40 units in the log of the ratio between the probability of being medium-risk vs. high-risk. Increasing frequent session depositing by one unit resulted in a decrease by -1.57 units in the log of the ratio between the probability of being medium-risk vs. high-risk.

**Table 3:** Coefficients of a multinomial model with risk score as dependent variable and chasing losses metrics as independent variables

Low Risk	Coefficient	Std. error	z	*p* > z
Constant	2.398	0.089	27.071	0.000
Within-session chasing	0.2117	0.143	1.518	0.129
Across-session chasing	0.1225	0.088	1.393	0.164
Across-days chasing	-0.0711	0.068	-1.044	0.296
Gambling account depletion	1.2324	0.129	9.519	0.000
Frequent session deposits	-7.4051	0.217	-34.203	0.000
Medium Risk				
Constant	0.9216	0.098	9.422	0.000
Within-session chasing	0.3464	0.156	2.218	0.027
Across-session chasing	0.0335	0.095	0.352	0.725
Across-days chasing	0.0328	0.074	0.445	0.656
Gambling account depletion	0.3969	0.142	2.794	0.005
Frequent session deposits	-1.5746	0.204	-7.71	0.000

**Fig. 1:**
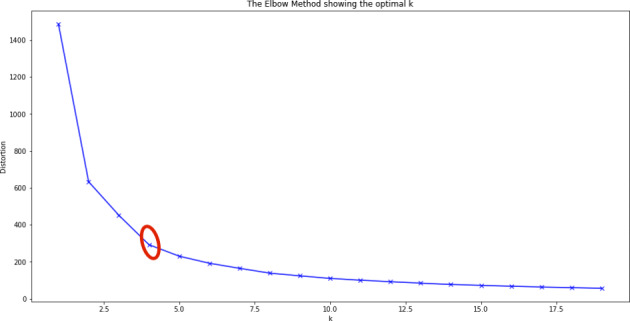
Elbow chart visualizing the optimal number of clusters for the given data set. The red circle indicates that four clusters are the best possible solution

In order to gain more insight into the behavioral patterns of the players, a k-means cluster analysis was performed. Only the two significant chasing losses metrics (i.e., regular gambling account depletion and frequent session depositing) were used. A z-score transformation was applied to the variables (Mohamad et al., 2013). After this standardization, each variable carried the same weight in the clustering process. The number of clusters was determined using the elbow method (Kaufmann & Rousseeuw, 2009). The elbow method is a visual approach which displays the within-sum of squares for different numbers of clusters. The optimal number of clusters appears at the so-called elbow where the slope changes most significantly. Figure 1 indicates that a four-cluster solution fitted the data best. The four-cluster datapoint is also indicated by the red circle in Fig. 1.

Table 4 reports the profile for each of the four identified clusters. The clusters are displayed in descending order according to size. Table 4 also reports the percentage of low-risk, medium-risk, and high-risk players in each cluster. The risk score was not used to cluster the data. However, there are differences with respect to the percentage of low-risk, medium-risk, and high-risk players between the four clusters. The largest percentage of high-risk (19%) and medium-risk players (34%) is in Cluster 4. Cluster 4 also had the lowest percentage of low-risk players (47%). A total of 10.35% of players are in Cluster 4. Cluster 4 also has the highest frequent session depositing value. Approximately 13% of players deposit on average more than once in their gambling sessions. The other three clusters frequent session depositing values are 4%. Cluster 4 also had the second highest gambling account depletion value. On average, 77% of those players sessions end with less than €5 in their gambling account.

**Table 4:** Mean average values for each of the four identified clusters

Cluster	N	Regular gambling account depletion	Frequent session depositing	Low-risk	Medium-risk	High-risk
Cluster 1	5102 (30.40%)	60%	4%	78%	16%	6%
Cluster 2	6817 (40.64%)	91%	4%	85%	12%	3%
Cluster 3	3116 (18.60%)	24%	4%	75%	18%	7%
Cluster 4	1736 (10.35%)	77%	13%	47%	34%	19%

## Discussion

Frequently used diagnostic instruments such as DSM-5 criteria for gambling disorder (American Psychiatric Association, 2013) as well as popular gambling screening instruments such as Problem Gambling Severity Index (Ferris & Wynne, 2001) contain a criterion for chasing losses. To the best of the authors’ knowledge, the present study is the first to ever compare different chasing losses metrics using online gambling account-based behavioral tracking data.

In the present study, five different chasing losses metrics were computed. Chasing losses was measured within gambling sessions, across gambling sessions, and across gambling days. Transaction data from a sample of 16,771 players from three countries (Spain, Sweden and the UK), provided by a large European online gambling operator were used to compute five chasing losses metrics. The average age of players was 41 years which is comparable to other player tracking studies which have used European online gambling operators’ data (e.g., Auer & Griffiths 2020; Hopfgartner et al., 2021). Half of the players were women which is a larger percentage of females compared to other player tracking studies which have used European online gambling operators’ data (Auer & Griffiths, 2020, 2021). One reason for the larger percentage of females could be the abundance of slot games with the present study’s online gambling operator. Research has found a preference for slots games among women compared to other types of gambling apart from bingo (LaPlante et al., 2006; Potenza et al., 2006, Baggio et al., 2018).

Just over 6% of the players were categorized as high-risk by the player tracking tool *mentor*. There was a significant difference with respect to age between the three gambling risk groups. However, the actual difference between the three gambling risk groups was less than one year and the significance was probably most likely due to the large sample size. The results indicated that 43% of high-risk players were female, 46% of medium-risk players were female and 53% of low-risk players were female. Studies have reported higher percentages of problem gambling among men (Merkouris et al., 2016) although the percentage of women in the high-risk category is higher than that reported in most prevalence surveys (Calado & Griffiths, 2016).

There was also a significant difference between the gambling-risk groups with respect to the number of gambling sessions, playing days, and number of deposits in the observation period. On average, high-risk players had 39 gambling sessions, medium-risk players had 29, and low-risk players had 21. On average, high-risk players had 15 playing days, medium-risk players had 14, and low-risk players had 11. On average, high-risk players had 25 monetary deposits, medium-risk players had 14, and low-risk players had four. These findings are in line with the ‘total consumption model’ (and applicable to many regulated commodities) (Sulkunen et al., 2012; Parker et al., 1978). The model predicts a high association between total population consumption and prevalence of excessive and problematic consumption (Lund, 2008). There is robust evidence supporting the applicability of the total consumption model to the gambling field (Rossow, 2019).

Within-session chasing, regular gambling account depletion, and frequent session depositing were significantly different between the three gambling risk groups. Although within-session chasing was significantly different between the three gambling risk groups, the actual values were very similar (i.e., the correlations were − 0.01 for low-risk gamblers, 0.01 for medium-risk gamblers and 0.00 for high-risk gamblers). Chasing would actually be reflected by negative correlations. More specifically, a negative correlation would indicate that larger losses in session are usually followed by larger bets in the next session. This is clearly not reflected in the results. There were no significant differences between the three gambling risk groups with respect to across-session chasing. There was a significant difference between three gambling risk groups with respect to across-days chasing. However, the correlation was positive in all three risk categories which indicates that low losses on a particular day are usually followed by larger bets on the next day.

Regular gambling account depletion indicates the percentage of sessions which terminate with no (or very little) money in the gambling account. Table 2 contradicts what was expected because high-risk players had the lowest percentage (64%) and low-risk players had the largest percentage (69%). Two possible conclusions can be drawn from such a finding. High-risk players either chase less frequently after their losses with respect to this particular operationalization, or the criterion does not reflect chasing losses. Given the large body of evidence that problematic players frequently chase losses, it is more likely that regular gambling account depletion is not an appropriate indicator of chasing losses.

Frequent session depositing which was used as a criterion of chasing losses by Challet-Bouju et al. (2020), had the largest percentage among high-risk players (18%). In the low-risk gambling group, players chased their losses according to this operationalization in only 5% of the sessions. The percentage was 13% among medium-risk players. The results suggest the importance of frequent sessions deposits as a proxy measure for chasing losses. Among the five operationalizations of chasing losses, only frequent session depositing had a meaningful and statistical difference between the three gambling risk groups. A multinomial regression supported the univariate findings with regular gambling account depletion and frequent session depositing being the only significant independent variables. The positive gambling account depletion coefficient in the multinomial regression for both low-risk and medium-risk player confirms the univariate findings that regular gambling account depletion was more likely among these two gambling groups. Therefore, it is concluded that regular gambling account depletion is not an appropriate indicator for chasing losses.

The negative frequent session depositing coefficient in the multinomial regression for both low-risk and medium-risk player confirms the univariate findings that frequent session depositing is less likely among these two groups. Therefore, it is concluded that frequent session depositing is an appropriate indicator for chasing losses. To gain further insights into subgroups of players, a k-means cluster analysis was performed. Using the elbow-method, four clusters were computed. Cluster 4 had the highest value with respect to frequent session depositing (13%) as well as the highest percentage of high-risk players (19%). Consequently, the percentages of low-risk and medium-risk players were lowest in Cluster 4. This exploratory result provides additional evidence that frequent session depositing reflected chasing losses better than any of the other four metric operationalizations used.

The present study has a number of limitations that are reflective of using behavior tracking data more generally. For instance, although the sample size is relatively large and the data are truly objective (which are both strengths of study), the data come from only one European gambling operator’s website and the players at this gambling website (who were from only three countries – Spain, Sweden and the UK) may not be representative of online gamblers more generally (or from other operators) and/or online gamblers from other countries. The data were also collected during one particular month (i.e., December) and gambling patterns may be different during other months. Moreover, the gambling operator’s portfolio of games may be specific to the website and may not be directly comparable with game portfolios at other gambling operators’ websites which may also have an impact on the findings (e.g., chasing losses on casino games is likely to be different from gambling operators who only offer lottery-type games). The only demographic data that was supplied by the gambling operator were the age and gender of the gamblers. Other demographic data may be useful in future studies (e.g., race/ethnicity). Also, there is always the possibility that some of the accounts were played by more than one individual (e.g., a married couple using the same account to gamble) although the number of such cases would likely be low. Moreover, the gambling risk ratings were determined by a behavioral tracking tool so it is not known whether these ratings were any more or less reliable than if self-report screening instruments had been used. Finally, the present study attempted to cover a wide range of chasing losses metrics. However, chasing losses could be measured in other ways the authors did not operationalize and/or contemplate.

## Conclusions

Findings from the present study were based on account-based player tracking data from European online casino gamblers. It is the first attempt in the gambling studies field to empirically compare different chasing losses metrics in an online casino setting. Depositing frequently in a gambling session was shown to be the most indicative of high-risk gambling. This is supported by previous research findings (e.g., Challet-Bouju et al., 2020; Perrot et al., 2018). The chasing losses metrics that were based on daily aggregated monetary spending were not correlated with high-risk gambling. The results underline the necessity for detailed player tracking procedures such as the identification of depositing frequently within sessions.

Many gambling operators are now routinely being asked by regulators in their jurisdictions to identify markers of harm related to problem gambling among their clientele by using behavioral analytics and to intervene and help protect players from developing problems. The present paper provides the gambling industry (as well as regulators and researchers who have access to gambling operators’ account-based tracking data) with five new metrics that may be indicative of chasing losses and which can be used as possible online markers of problem gambling harm. Regulatory bodies could also use the findings of this study to contemplate new player safety features such as a maximum number of deposits per gambling session. Based on the findings here, it is not sufficient for gambling operators to simply use daily aggregated data to identify problematic gambling. However, although frequent session depositing appears to be more indicative of chasing losses than the other four metrics, all the metrics provide useful information which can be used to help identify problematic gambling behavior online.

## Footnotes

1 corr_i,s_ is the correlation between the amount of money wagered and time for player i in session s. w_i,s_ is the vector of wagers for player i in session s. t_i,s_ is the vector of consecutive numbers beginning with the first wager to the last wager for player i and session s.

2 corr_i,_ is the average correlation computed across all sessions for player i.

3 corr_i_ (l_i,t−1_,w_i,t_) is the average correlation between the loss of money in session t-1 and amount of money wagered in the consecutive session t for player i.
